# Clinical Features of COVID-19 Patients with Diabetes and Secondary Hyperglycemia

**DOI:** 10.1155/2020/3918723

**Published:** 2020-08-24

**Authors:** Wan Zhou, Shandong Ye, Wei Wang, Sumei Li, Qinggang Hu

**Affiliations:** ^1^Department of Endocrinology, The First Affiliated Hospital of USTC, Division of Life Sciences and Medicine, University of Science and Technology of China, Hefei, China; ^2^Department of Infectious Diseases, The First Affiliated Hospital of USTC, Division of Life Sciences and Medicine University of Science and Technology of China, Hefei, China

## Abstract

People with diabetes have higher risks of various infections. Therefore, these diabetic patients might be at increased risk of COVID-19 and have a poorer prognosis. Up until now, little is known about critical role in the pathogenesis. This study aims to investigate the clinical characteristics of COVID-19 patients with diabetes and secondary hyperglycemia, as well as to explore the purported mechanisms. 80 confirmed COVID-19 subjects were classified into the euglycemia group, secondary hyperglycemia group, and diabetes group. Severity of COVID-19 was defined based on the diagnostic and treatment guideline for SARS-CoV-2 issued by Chinese National Health Committee. According to the severity of the disease, patients of the mild type and common type were registered as mild cases (patients with minimal symptoms and negative CT findings), while patients of the severe type and critical type were enrolled as severe cases (patients with positive CT findings and different extent of clinical manifestations). Patients in the diabetes group were older than those in the euglycemia group, and most of them were male. In the diabetes group, the proportion of severe cases was 57.14%, which was significantly higher than those in the other two groups, and 32% of the COVID-19 patients diagnosed as severe cases were with diabetes. The CD4+ cell counts in the diabetes group were lower than those in the other two groups, while the levels of LDH and hs-CRP were higher. Compared with the euglycemia group, the CD3+ cell counts and the CD4+/CD8+ ratio were decreased, whereas the levels of IL-6 were increased in the secondary hyperglycemia group and diabetes group, with the diversities in the diabetes group being especially more significant. The Spearman correlation analysis revealed that the presence of diabetes was positively correlated with age, hs-CRP, LDH, IL-6, CD8+ cells, and severity of COVID-19 and negatively correlated with CD3+ cell counts, CD4+ cell counts, and CD4+/CD8+ ratio. Compared with the other two groups, the diabetes group exhibited more diverse and multifocal features in CT imagings. Diabetes is a risk factor for influence of the progression and prognosis of COVID-19 due to ongoing inflammation and impaired immune response.

## 1. Introduction

In December 2019, the pneumonia caused by a novel coronavirus infection erupted in Wuhan, Hubei Province [1]. Up to April 10^th^ 2020, there have been more than 1.6 million confirmed cases of 2019 novel coronavirus disease (COVID-19) worldwide, accounting for 95,559 deaths, which has become the focus of global attention. The person-to-person transmission routes of COVID-19 comprised of direct transmission, such as through coughing, sneezing, contact transmission, and droplet inhalation transmission. COVID-19 is characterized by quick onset, strong infectivity. and high incidence of the susceptibility crowd, whose symptoms may include fever, fatigue, cough, dyspnea, and tightness [2]. For the majority of cases, these symptoms are minor; but for a few, this viral infection can lead to pneumonia and multiorgan failure.

Diabetes mellitus (DM) is a common chronic metabolic disease. With the improvement of people's living standards, the prevalence rate of diabetes keeps rising on a yearly basis. Diabetes patients have higher risks of contracting various infections which might be accounted for by multiple complications and compromised immunity. As portrayed by previous data, while diabetes cooccurred, the odds ratio of death or severe complications following middle east respiratory syndrome coronavirus (MERS-CoV) infection ranged from 2.47 to 7.24 [3], and the risk ratio of poor outcome (death, ICU admission, or mechanical ventilation) for the severe acute respiratory syndrome (SARS) patients was higher (3.1, 95% CI, 1.4-7.2) than for those without diabetes [4]. Therefore, diabetes was found to be a host-independent risk factor for mortality and morbidity in patients with MERS or SARS [5, 6].

Up to now, whether or not patients with diabetes have a higher susceptibility to COVID-19 is still not clear. However, there is a perception that the risks of infection and severe disease are both higher in patients with diabetes. A meta-analysis including 1,527 patients with COVID-19 reported that the prevalence of diabetes was 9.7%, and the incidence of diabetes in severe cases was about twice than that in nonsevere counterparts [7]. According to an early analysis of a small cohort in Wuhan [8], diabetes accounted for approximately 20% of the intensive care unit (ICU) admission. More recent data from Italy showed more than two-thirds of those who died by COVID-19 had diabetes [9]. Consequently, exploring the clinical characteristics of COVID-19 patients with diabetes will conduce to a reduction in the incidence of severity. We performed a single-center retrospective study of the confirmed COVID-19 cases consisting of 80 cases to discuss the correlation between diabetes, secondary hyperglycemia, and COVID-19, together with the possible responding mechanisms.

## 2. Materials and Methods

According to the diagnosis and treatment criteria of COVID-19 issued by China National Health Commission, 80 patients were hospitalized in Anhui Provincial Hospital from January to March 2020.

### 2.1. Clinical Classifications

Clinical classifications of COVID-19 were defined based on the diagnostic and treatment guideline for severe acute respiratory syndrome coronavirus 2 (SARS-CoV-2) issued by Chinese National Health Committee (Version 6) as follows: (1) mild type: mild clinical symptoms without pneumonia in imaging. (2) Common type: fever and respiratory symptoms with pneumonia in imaging. (3) Severe type: respiratory distress; respiratory rate ≥30 times/min; oxygen saturation ≤93% in resting state; PaO2/FiO2 ≤300 mmHg; imaging showed significant progression which was above 50% within 24-48 hours. (4) Critical type: the patients fitted any of the following circumstances: (1) respiratory failure requiring mechanical ventilation; (2) shock and other organ failure; (3) requiring ICU monitoring and treatment.

### 2.2. Grouping

Based on their blood glucose levels, the patients were assigned into 3 groups. (1) The euglycemia group: there were 44 patients composed of 21 males and 23 females, all of whom had no histories of diabetes, and the range of their age was 27-52 years old. (2) The secondary hyperglycemia group: there were 22 patients consisting of 17 males and 5 females, who met the conditions of no past histories of diabetes, hemoglobin A1c (HbA1c) <6.5%, random blood glucose >11.1 mmol/L during hospitalization, and normal blood glucose after discharge from the hospital. The range of their age was 40-70 years old, and 5 patients among them had elevated blood sugar after glucocorticoid therapy. (3) The diabetes group: there were 14 patients including 10 males and 4 females, all of whom were type 2 diabetes mellitus (T2DM) patients. And they were treated with oral antidiabetics or insulin before hospitalization and without glucocorticoid therapy during hospitalization, their ages ranged from 43 to 67 years old. Besides, according to the severity of the disease and clinical classifications, patients of the mild type and common type were registered as mild cases (*n* = 55), while patients of the severe type and critical type were enrolled as severe cases (*n* = 25).

### 2.3. Method

Patient informations, for instance, demographic data, epidemiology, clinical symptoms, laboratory examinations, and chest computed tomography (CT) findings, were collected, in which clinical features included fever, fatigue, cough, chest tightness, dyspnea, diarrhea, and other symptoms, and laboratory examination included peripheral leukocyte count, lymphocyte absolute value, neutrophils absolute value, lymphocyte percentage, neutrophils percentage, high-sensitive C-reactive protein (hs-CRP), procalcitonin (PCT), erythrocyte sedimentation rate (ESR), alanine aminotransferase (ALT), lactate dehydrogenase (LDH), alkaline phosphatase (ALP), aspartate aminotransferase (AST), total bilirubin (TB), gamma-glutamyltransferase (*γ*-GGT), D-dimer, creatine kinase (CK), creatine kinase isoenzyme-MB (CK-MB), prothrombin time (PT), highly sensitive troponin I (hs-TnI), activated partial thromboplastin time (APTT), the phenotypic analysis of lymphocytes (CD4+, CD8+, CD3+ T cells), and interleukin-6(IL-6).

### 2.4. Statistical Analyzes

SPSS 22.0 statistical software (IBM, Armonk, NY, USA) was used for statistical analysis of the data. Medians and interquartile ranges (IQRs) were calculated as summaries of continuous variables. Characteristics were assessed among three subgroups by the Kruskal-Wallis test and chi-squared test. Spearman's rank correlation analysis was applied to evaluate the correlation of two characteristics. *P* < 0.05 was considered statistically significant.

## 3. Results

### 3.1. Epidemiological, Demographic and Basic Informations

As shown in Table 1, the median age of the 80 hospitalized COVID-19 patients was 47 years old [(interquartile range (IQR), 35-56)], and 48 cases (80%) were men. No death happened in our cohort. In terms of onset symptoms, 68 (85%) patients exhibited symptoms of fever, while 30 (37.5%) had symptoms of fatigue, the other two common symptoms were cough (56.25%) and chest tightness (33.75%), whereas diarrhea (11.39%) and dyspnea (10%) were relatively rare. As illustrated in Table 2, there were 14 COVID-19 patients complicated with diabetes (17.5%), 22 patients with secondary hyperglycemia (27.5%), and 44 patients with normal glucose levels (55%). The proportion of severe cases was higher in the diabetes group (57.14%) compared with the other two groups. Meanwhile, the average age in the diabetes group was higher than that in the other two groups (*P* < 0.05). The proportion of males in the diabetes group and secondary hyperglycemia group was more elevated than that in euglycemia group.

### 3.2. Laboratory Findings

**Figure 1:**
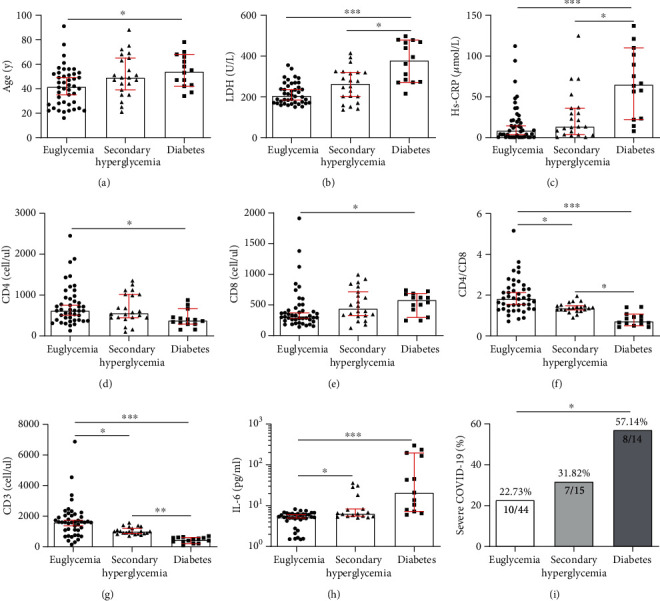
Comparison of laboratory findings in each group, ^∗^*P* < 0.05; ^∗∗^*P* < 0.01; ^∗∗∗^*P* < 0.001. Hs-CRP: high-sensitive C-reactive protein; IL-6: interleukin-6.

As displayed in Table 2 and Figure 1, raised LDH levels as well as higher concentrations of hs-CRP were present in the diabetes group compared with the secondary hyperglycemia group [LDH 378 (272.50-470.50) vs 264 (194.00-321.00), hs-CRP 65.05 (23.15-103.68) vs 13.55 (3.83-36.43)] and euglycemia group [LDH 378 (272.50-470.50) vs 205.50 (171.00-256.25), hs-CRP 65.05 (23.15-103.68) vs 8.70 (0.60-21.93)] (*P* < 0.05). Analysis of cellular immunity also revealed several discrepancies, notably regarding CD3+ cells, CD4+ cell counts, CD8+ cell counts, and CD4+/CD8+ ratio. Specifically, the number of CD4+ cells was decreased in the diabetes group when compared to secondary hyperglycemia group [376.5 (290.63-509.75) vs 556 (446.00-1015.75)] and euglycemia group [376.5 (290.63-509.75) vs 618.5 (88.75-928.00)] (*P* < 0.05); the number of CD3+ cells and the ratio of CD4+/CD8+ were lower in the diabetes group [CD3+ cells 446.50 (311.00-593.50) vs 1604.50 (1086.25-2043.00), CD4+/CD8+ 0.72 (0.57-1.00) vs 1.82 (1.32-2.35)] and secondary hyperglycemia group [CD3+ cells 1002 (868.25-1230.75 vs 1604.50 (1086.25-2043.00), CD4+/CD8+ 1.36 (1.26-1.49) vs 1.82 (1.32-2.35)] than that in the euglycemia group, with the decrease in the diabetes group being particularly more significant. (*P* < 0.05). Moreover, with respect to the inflammatory mediators, IL-6 concentrations in the euglycemia group, secondary hyperglycemia group, and diabetes group were, respectively, [5.26 (0.10-0.43)], [6.39 (0.19-0.45)], and [32.11 (0.19-0.67)], which indicates that the level of IL-6 was much higher in diabetes group. No considerable differences in other clinical characteristics, such as incubation period, onset symptoms, lymphocyte percentage, neutrophils percentage, PCT, creatinine, urea nitrogen, ALT, AST, ALP, *γ*-GGT, TB, ESR, D-dimer, CK, CK-MB, hs-TnI, PT, and APTT, were observed across the three groups.

### 3.3. The Proportion of COVID-19 Patients with Diabetes

**Figure 2:**
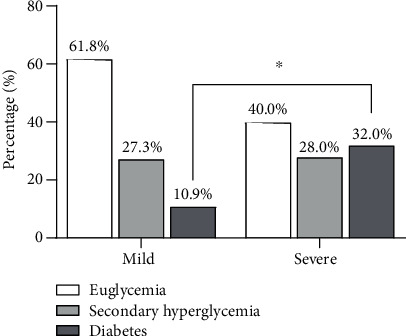
The corresponding proportions in each group. Mile cases: patients of the mild type and common type; severe cases: patients of the severe type and critical type.

32% of COVID-19 severe cases were patients with diabetes. In contrast, only 10.9% of mild cases had diabetes (*P* < 0.05). The corresponding proportions are exhibited in Figure 2.

### 3.4. CT Imagings

**Figure 3:**
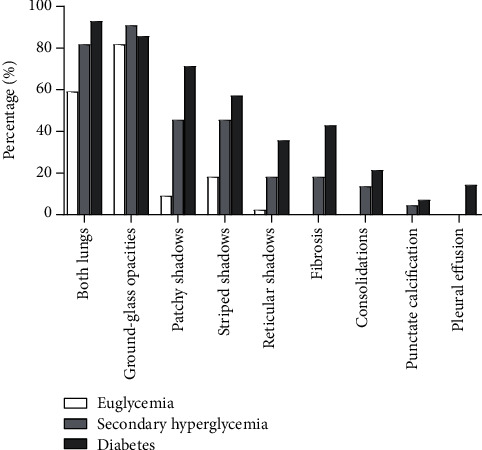
The frequency of evaluated CT imaging features in three groups.

**Figure 4:**
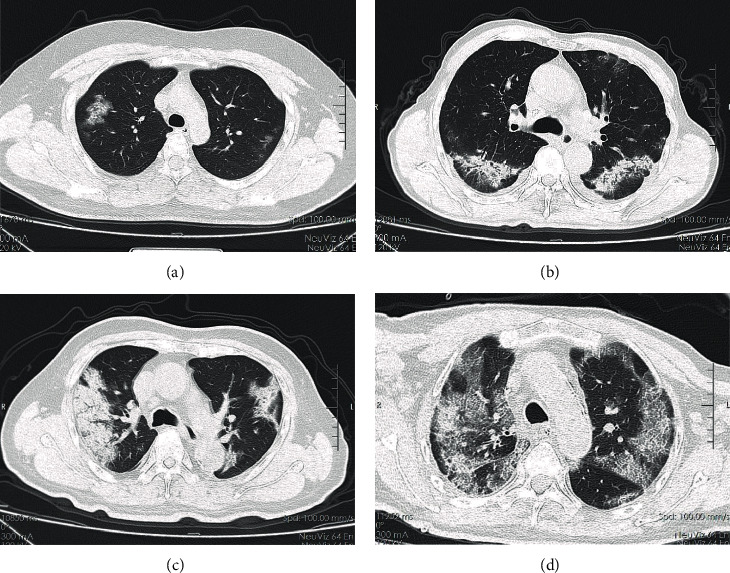
Typical CT images of the patients in three groups: (a) from euglycemia group; (b) from secondary hyperglycemia; (c, d) from diabetes group.

78 cases (97.5%) had evidence of pneumonia on CT among the 80 COVID-19 patients, and the corresponding proportions in the euglycemia group, secondary hyperglycemia group, and diabetes group were 95.5%, 100%, and 100.0%, respectively. Additionally, the lesions were distributed in both lungs for most patients (83.8%), the corresponding proportion in the diabetes group was 92.9%, which was higher than that in the other two groups. Furthermore, there were 68 cases with ground-glass opacities, 24 cases with patchy shadows, 26 cases with striped shadows, 10 cases with reticular shadows, 10 cases with fibrosis, 6 cases with consolidations, 2 cases with punctate calcification, and 2 cases with pleural effusion. The frequency of evaluated CT imaging features in three groups are delineated in Figure 3, and typical CT imagings are shown in Figure 4. It can be seen that compared with the other two groups, the diabetes group contained more diverse and multifocal features.

### 3.5. Spearman's Correlation Analysis

As depicted in Tables 3 and 4, the Spearman correlation analysis results revealed that the presence of diabetes was positively correlated with age (*r* = 0.346), hs-CRP (*r* = 0.556), LDH (*r* = 0.635), IL-6 (*r* = 0.703), CD8+ (*r* = 0.332), severity of COVID-19 (*r* = 0.318) and negatively correlated with CD3+ (*r* = −0.638), CD4+ (*r* = −0.351), CD4+/CD8+ (*r* = −0.652), and the presence of secondary hyperglycemia was positively associated with IL-6 (*r* = 0.411) and inversely correlated with CD3+ (*r* = −0.388) and CD4+ (*r* = −0.391).

## 4. Discussion

COVID-19 is a contagious disease characterized by high infectivity and mortality, especially for patients with severe underlying diseases such as diabetes. In this study, 17.5% of COVID-19 patients also had diabetes, which was consistent with the previous reports in which the proportion of COVID-19 patients with diabetes ranged from 10.1% to 20% [10–12], and the proportion of COVID-19 severe cases with diabetes was 32%, which was higher than the data (16.2%) reported by Shahid et al. [13]. As a severe acute respiratory syndrome-associated coronavirus (SARS-CoV) receptor, the angiotensin-converting enzyme 2 (ACE2) receptor, which is widely distributed in the heart, kidney, intestine, etc., is also considered as a key receptor for COVID-19. The SARS-CoV-2 is able to bind to ACE2 receptors located in the cell membrane of respiratory and lung epithelial cells, causing respiratory infections, as well as the ones in the islets leading to islet cell injury and raised blood glucose [10]. Secondary hyperglycemia is commonly associated with a variety of nondiabetic conditions such as hormonal abnormalities, drug side effects, and stress state. There were 22 patients in the secondary hyperglycemia group in this study, 17 of whom were not treated with glucocorticoid during hospitalization, which implied that besides acute stress factors, the pneumonia virus infection may also be one of the causes of hyperglycemia. New-onset diabetes and severe metabolic complications of preexisting diabetes have been observed in patients with COVID-19 in the recent studies [14, 15], and these observations provide support for the hypothesis of a potential diabetogenic effect of COVID-19, beyond the well-recognized stress response associated with severe illness [16]. Similar to what has been described with previous pandemic of SARS-CoV, greater incidences of fasting glycemia and acute onset diabetes have been reported among patients with SARS coronavirus 1 pneumonia than among those with non-SARS pneumonia [17]. Hyperglycemia was an independent predictor of death, and patients with even mild SARS (receiving no glucocorticoid medications during the course) had a higher level of fasting glycemia [18]. High and aberrantly glycated ACE2 in the tissues in uncontrolled hyperglycemia might favor the cellular intrusion of SARS-CoV-2, leading to a higher propensity to COVID-19 infection as well as a higher disease severity [19]. Due to the SARS-CoV-2, the pancreatic damage and the resultant impairment in *β*-cell insulin secretion may worsen preexisting diabetes or determine the appearance of hyperglycemia in nondiabetes [20].

This study indicated that as one of the indexes for assessing the severity of lung injury, the level of LDH was significantly higher in COVID-19 patients with diabetes than in the control group. In addition, the bilateral pulmonary lesions of COVID-19 patients in the diabetes group had a wider distribution than those in the other groups. From the view of grouping, both the proportion of severe patients in the diabetes group and the proportion of diabetes in severe cases increased, which indicated that COVID-19 patients with diabetes were more severe. The specific mechanism may be related to age, low immune function, and inflammatory mediation. In our study, patients in the diabetes group were older than those in other groups, which might be related to the fact that older patients have poorer abilities to fight off infections, more enhanced susceptibility to infection and more serious clinical symptoms after being infected by the SARS-CoV-2. Furthermore, abnormal T cell subsets, such as the decreases of CD3+ cell counts, CD4+ cell counts, and CD4+/CD8+ ratio, were observed in the diabetes group compared with the euglycemia group, and the presence of diabetes and secondary hyperglycemia were negatively correlated with CD3+ cell counts and CD4+/CD8+ ratio, which implies that the immune function of these patients was compromised. Immune dysfunction in infected diabetes patients revealed activated NK cells, increased inflammatory cytokines, reduced T-helper (Th)-associated chemokine receptors, and impaired T-cell proliferation at the onset of the disease [21]. Meanwhile, glycation of immunoglobulin occurring in diabetes patients may harm the biological function of the antibodies and finally lead to decrease in immunity with the increase of HbA1c [22]. When people were infected by SARS-COV-2, the decrease of insulin secretion and the worsening of insulin resistance may induce hyperglycemia, with immunoreaction and inflammatory activation, which, in turn, may further damage *β*-cells and worsen insulin resistance [23].

Fu [24] has proven that the SARS-CoV-2 mediated the innate immune system by downregulating and shedding virus-induced ACE2, which causes the release of large amounts of cytokines in the body. The principal physiological and pathological characteristic of severe COVID-19 infection is the “cytokine storm,” also known as “inflammatory storm” [2], which is due to the positive feedback loop between cytokines and immune cells. In other words, the immune system evolves from a “self-protective” state to an “overprotective” state, thus causing pulmonary edema and consolidation, systemic capillary bleeding, hypotensive shock, and multiple organ failure in severe cases. In the present study, the levels of inflammation indexes in diabetes group and secondary hyperglycemia group were both abnormal, and the diversities in diabetes group were even more significant. Meanwhile, the presence of diabetes was positively associated with hs-CRP and IL-6 in the COVID-19 patients. An aetiopathological role of inflammation in the development and progression of T2DM is increasingly accepted [25, 26], inflammatory cytokines can cause both structural and functional abnormities in endothelial cells leading to the dysfunction of insulin secretion and the damage of islet *β* cells, which will eventually result in an increase in blood glucose [27]. Patients infected with SARS-CoV-2 demonstrated abnormal expressions of cytokines, including interleukin-1 (IL-1), IL-6, interferon-*γ* (IFN-*γ*), and tumor necrosis factor-*α* (TNF-*α*) in the serum at early stages of the disease, which were higher in severe cases than in mild cases [28]. The serum levels of IL-6 and CRP can effectively evaluate the severity and predict the prognosis in patients with COVID-19 [29]. IL-6 is a class of multifunctional proteins produced essentially by macrophage and TH2 cells, which can participate in the anti-infectious and autoimmune functions of the body by inducing the proliferation of B lymphocytes, as well as promoting the proliferation and activation of T lymphocytes. It is an important predictor of disease severity and prognosis, and its expression time is longer than that of other cytokines such as IL-1 and TNF-*α* [30]. Hyperglycemia and diabetes are linked with chronic low-grade inflammatory processes and related vulnerability to infection, which can impair antiviral activity and increase the risk for fatality [31]. Chronic inflammation perhaps acts as the underlying mechanism enhancing a cytokine storm hyperinflammatory state that triggers multiorgan failure in COVID-19-infected individuals [32]. Therefore, patients with T2DM are characterized by a pervasive status of low-grade inflammation coupled by a progressive decline of immune system function evidenced by abnormal T cell subsets. These two major characteristics predispose subject with T2DM to an exaggerated cytokine storm, coupled with “immune paralysis,” which in turn seems to be responsible for the high fatality rate observed in T2DM patients infected with SARS-CoV-2.

Above all, diabetes is associated with increased incidence and severity of COVID-19, and more intensive attention should be paid to diabetes patients in order to prevent rapid deterioration, since hypoxemia and abnormal liver and kidney functions exist in most COVID-19 patients, oral hypoglycemic drugs (especially biguanidine) should not be administered, while insulin injection, intravenous drips, or insulin pumps are recommended to be used frequently. On this basis, diet restriction can be relaxed appropriately to enhance the resistance to infection of patients with diabetes.

In a nutshell, diabetes patients appear to be at higher risk of severe illness from COVID-19 than those without diabetes due to ongoing inflammation, impaired immune response, which entails that they are more likely to have worse prognoses. As a result, meticulous attention should be paid to the management of blood glucose in COVID-19 patients with diabetes, and anti-inflammatory therapy combined with immunomodulation may be considered as one of the effective treatment methods. There were several limitations in our study. First of all, this is a single-center and retrospective analysis, the sample size is relatively small, and the characteristics of COVID-19 patients with diabetes could not be fully summarized. Secondly, some important laboratory test results, such as those regarding the inflammatory factors (IFN-*γ*, TNF-*α*), were not analyzed in our study due to the limited data availability. Thirdly, there were no type 1 diabetes mellitus (T1DM) patients in our cohort. Thence, further research is expected to be carried out with more comprehensive information acquisition.

## Figures and Tables

**Table 1:** The basic informations of the patients with COVID-19 (*n* = 80).

Characteristics	P_50_ (P_25_ − P_75_), *n* (%)
Age (*y*)	47.00 (35.00-56.00)
Gender	
Male	48 (60.00)
Female	32 (40.00)
Severe cases (*n*, %)	55 (68.75)
Mild cases (*n*, %)	25 (31.25)
Symptoms	
Fever (*n*, %)	68 (85.00)
Fatigue (*n*, %)	30 (37.5)
Cough (*n*, %)	45 (56.25)
Chest tightness (*n*, %)	27 (33.75)
Dyspnea (*n*, %)	8 (10.00)
Diarrhea (*n*, %)	9 (11.39)
Incubation (d)	6.00 (4.00-9.00)
Hs-CRP (*μ*mol/L)	14.00 (3.80-41.60)
Leukocyte (10 × 9/L)	5.34 (4.12-6.61)
Platelet (10 × 9/L)	163.00 (129.00-213.50)
Lymphocyte percentage (%)	21.10 (10.83-31.20)
Neutrophils percentage (%)	70.15 (59.05-80.05)
Lymphocyte (10 × 9/L)	1.06 (0.67-1.49)
Neutrophil (10 × 9/L)	3.57 (2.30-5.25)
Calcitonin zymogen (ng/ml)	0.14 (0.10-0.18)
ESR (mm/h)	45.00 (16.50-65.75)
LDH (U/L)	237.00 (182.50-302.00)
Creatinine (*μ*mol/L)	69.00 (59.25-80.00)
Urea nitrogen (mmol/L)	4.06 (2.99-5.60)
ALT (IU/L)	24.00 (15.00-42.00)
AST (IU/L)	26.50 (21.00-36.75)
ALP (IU/L)	59.50 (46.00-71.00)
*γ*-GGT (IU/L)	29.50 (18.50-50.25)
TB (*μ*mol/L)	14.50 (11.10-18.85)
CD4/CD8	1.42 (1.19-1.91)
CD4 (cell/*μ*l)	553.00 (383.50-869.75)
CD8 (cell/*μ*l)	367.17 (266.54-617.98)
CD3 (cell/*μ*l)	1086.50 (687.50-1625.25)
IL-6 (pg/ml)	6.13 (5.11-7.98)
D-dimer (*μ*g/ml)	0.24 (0.14-0.45)
CK (IU/L)	86.70 (52.65-141.03)
CK-MB (U/L)	10.95 (8.68-15.10)
hs-Tn (*μ*g/L)	0.09 (0.06-0.27)
PT(s)	14.45 (13.48-16.00)
APTT(s)	36.50 (33.48-41.10)

Data are shown as medians and interquartile ranges. Hs-CRP: high-sensitive C-reactive protein; PCT: procalcitonin; ESR: erythrocyte sedimentation rate; LDH: lactate dehydrogenase; ALT: alanine aminotransferase; AST: aspartate aminotransferase; ALP: alkaline phosphatase; *γ*-GGT: gamma-glutamyltran; TB: total bilirubin; IL-6: interleukin-6; CK: creatine kinase; CK-MB: creatine kinase isoenzyme-MB; hs-TnI: highly sensitive troponin I; PT: prothrombin time; APTT: activated partial thromboplastin time.

**Table 2:** Comparison of laboratory findings in three groups.

Characteristics	Euglycemia (*n* = 44)	Secondary hyperglycemia (*n* = 22)	Diabetes (*n* = 14)	*Z*/*χ*^2^	*P*
Age (*y*)	41.50 (27.25-52.00)	49.00 (38.00-65.75)	54.00 (42.75-66.50)^a^	8.071	0.018
Gender				6.258	0.044
Male	21 (47.73)	17 (77.27)^a^	10 (71.43)^a^		
Female	23 (52.27)	5 (22.73)^a^	4 (28.57)^a^		
Severe				5.348	0.021
No	34 (77.27)	15 (68.18)	6 (42.86)^a^		
Yes	10 (22.73)	7 (31.82)	8 (57.14)^a^		
Symptoms					
Fever (*n*, %)	38 (86.3)	18 (81.8)	12 (85.7)	0.244	0.885
Fatigue (*n*, %)	14 (31.8)	8 (36.3)	8 (57.1)	2.923	0.232
Cough (*n*, %)	25 (56.8)	12 (54.5)	8 (57.1)	0.036	0.982
Chest tightness (*n*, %)	14 (31.8)	8 (36.3)	5 (35.7)	0.165	0.921
Dyspnea (*n*, %)	4 (9.09)	2 (9.09)	2 (14.2)	0.346	0.841
Diarrhea (*n*, %)	4 (9.09)	3 (13.64)	2 (14.2)	0.460	0.794
Incubation (d)	5.00 (4.00-7.00)	6.50 (3.75-10.00)	6.50 (4.75-10.00)	3.426	0.180
Hs-CRP (*μ*mol/L)	8.70 (0.60-21.93)	13.55 (3.83-36.43)	65.05 (23.15-103.68)^a,b^	18.963	0.000
Leukocyte (10 × 9/L)	5.30 (3.80-6.51)	5.12 (4.57-6.37)	6.58 (4.35-7.50)	3.068	0.216
Platelet (10 × 9/L)	163.50 (123.75-216.00)	171.00 (142.50-204.50)	143.00 (119.00-211.50)	0.588	0.745
Lymphocyte percentage	21.35 (12.73-34.90)	21.55 (13.53-28.00)	14.70 (7.40-26.35)	3.026	0.220
Neutrophils percentage	68.90 (52.50-77.90)	70.15 (63.58-80.85)	77.80 (63.50-86.40)	4.867	0.088
Lymphocyte (10 × 9/L)	1.08 (0.69-1.64)	1.09 (0.57-1.48)	1.00 (0.53-1.19)	1.663	0.435
Neutrophil (10 × 9/L)	2.87 (2.08-4.75)	3.52 (3.05-4.72)	5.37 (3.14-6.37)	4.221	0.121
PCT (ng/ml)	0.14 (0.10-0.18)	0.14 (0.10-0.19)	0.16 (0.10-0.20)	0.455	0.797
ESR (mm/h)	29.10 (13.50-66.50)	34.00 (14.00-54.90)	66.00 (32.90-88.50)	4.423	0.110
LDH (U/L)	205.50 (171.00-256.25)	264.00 (194.00-321.00)	378.00 (272.50-470.50)^a,b^	22.642	0.000
Creatinine (*μ*mol/L)	68.50 (57.25-79.75)	69.50 (59.75-81.25)	70.00 (59.25-75.50)	0.139	0.933
Urea nitrogen (mmol/L)	3.79 (2.94-4.58)	4.42 (3.16-5.72)	5.24 (2.96-6.60)	4.448	0.108
ALT (IU/L)	20.00 (13.00-42.25)	29.50 (20.50-47.25)	24.50 (15.50-34.50)	4.526	0.104
AST (IU/L)	26.00 (20.00-35.50)	27.50 (21.75-46.50)	26.50 (20.75-33.00)	1.706	0.426
ALP (IU/L)	62.50 (49.00-71.75)	51.00 (41.00-71.00)	51.50 (46.50-71.75)	0.988	0.610
*γ*-GGT (IU/L)	24.50 (17.00-46.00)	33.00 (24.50-53.25)	44.50 (19.25-64.50)	5.018	0.081
TB (*μ*mol/L)	14.90 (11.73-18.58)	13.45 (9.78-19.40)	12.50 (8.58-22.25)	0.344	0.842
CD4 (cell/ul)	618.50 (388.75-928.00)	556.00 (446.00-1015.75)	376.50 (290.63-509.75)^a^	7.927	0.019
CD8 (cell/*μ*l)	314.16 (246.16-441.16)	440.36 (324.75-735.06)	580.16 (391.88-679.93)^a^	7.858	0.020
CD3 (cell/*μ*l)	1604.50 (1086.25-2043.00)	1002.00 (868.25-1230.75)^a^	446.50 (311.00-593.50)^a,b^	34.021	0.000
CD4/CD8	1.82 (1.32-2.35)	1.36 (1.26-1.49)^a^	0.72 (0.57-1.00)^a,b^	32.113	0.000
IL-6 (pg/ml)	5.26 (4.41-6.30)	6.39 (5.51-13.32)^a^	32.11 (7.80-206.73)^a,b^	29.867	0.000
D-dimer (*μ*g/ml)	0.21 (0.10-0.43)	0.25 (0.19-0.45)	0.37 (0.19-0.67)	3.328	0.189
CK (IU/L)	75.90 (54.50-138.90)	104.60 (50.43-205.00)	80.40 (39.00-178.30)	1.374	0.503
CK-MB (U/L)	10.50 (8.70-14.90)	11.30 (9.58-15.50)	9.20 (6.95-15.65)	1.749	0.417
hs-TnI (*μ*g/L)	0.10 (0.06-0.28)	0.09 (0.04-0.18)	0.09 (0.08-0.64)	2.777	0.249
PT (s)	14.60 (13.75-16.63)	14.25 (13.40-15.18)	14.05 (12.78-14.63)	4.914	0.086
APTT (s)	37.30 (34.43-41.40)	35.05 (32.25-39.83)	35.50 (32.18-41.58)	1.868	0.393

Data are shown as medians and interquartile ranges. The euglycemia group: the patients had no histories of diabetes. The secondary hyperglycemia group: the patients met the conditions of no past histories of diabetes, hemoglobin A1c (HbA1c) <6.5%, fasting blood glucose >6.1 mmol/L, and normal blood glucose after discharge from the hospital. The diabetes group: the patients had past histories of type 2 diabetes mellitus. Hs-CRP: high-sensitive C-reactive protein; PCT: procalcitonin; ESR: erythrocyte sedimentation rate; LDH: lactate dehydrogenase; ALT: alanine aminotransferase; AST: aspartate aminotransferase; ALP: alkaline phosphatase; *γ*-GGT: gamma-glutamyltran; TB: total bilirubin; IL-6: interleukin-6; CK: creatine kinase; CK-MB: creatine kinase isoenzyme-MB; hs-TnI: highly sensitive troponin I; PT: prothrombin time; APTT: activated partial thromboplastin time. Note: compared with euglycemia group, aP <0.05; compared with secondary hyperglycemia group, bP <0.05, in which *P* < 0.05 was considered statistically significant.

**Table 3:** Spearman's correlation between the presence of diabetes and different characteristics.

Characteristics	Correlation coefficient	*P*
Age (*y*)	0.346	0.008
Severe COVID-19	0.318	0.015
Hs-CRP (*μ*mol/L)	0.556	0.000
LDH (U/L)	0.635	0.000
CD4 (cell/*μ*l)	-0.351	0.007
CD8 (cell/*μ*l)	0.332	0.011
CD4/CD8	-0.652	0.000
CD3 (cell/*μ*l)	-0.638	0.000
IL-6 (pg/ml)	0.703	0.000

Hs-CRP: high-sensitive C-reactive protein; LDH: lactate dehydrogenase; IL-6: interleukin-6. The presence of diabetes was positively correlated with age, hs-CRP, LDH, IL-6, CD8+, severity of COVID-19, and negatively correlated with CD3+, CD4+, CD4+/CD8+.

**Table 4:** Spearman's correlation between the presence of secondary hyperglycemia and different characteristics.

Characteristics	Correlation coefficient	*P*
CD4/CD8	-0.391	0.001
CD3 (cell/*μ*l)	-0.388	0.001
IL-6 (pg/ml)	0.411	0.002

IL-6: interleukin-6. The presence of secondary hyperglycemia was positively associated with IL-6 and inversely correlated with CD3+ and CD4+.

## Data Availability

The data used to support the findings of this study are available from the corresponding author upon request.
